# A Case Report of Glycogenic Hepatopathy

**DOI:** 10.21980/J8SQ0Z

**Published:** 2021-07-15

**Authors:** Dane Brown, Theresa Mead

**Affiliations:** * Central Michigan University College of Medicine, Department of Emergency Medicine, Mt Pleasant, MI

## Abstract

Glycogenic hepatopathy is a rare condition that may be seen in patients with poorly controlled diabetes mellitus. This is a case presentation of a 15-year-old female with type 1 diabetes mellitus who was not fully adherent to her insulin regimen and presented in acute diabetic ketoacidosis. She had the associated symptom of two months of abdominal pain. The patient’s ketoacidosis resolved during her hospitalization with insulin and intravenous fluid management; however, her lactic acid and liver transaminases remained persistently elevated. Abdominal ultrasound was consistent with parenchymal liver disease and a clinical diagnosis of glycogenic hepatopathy was made. Management consisted of tight glucose control. After discharge, the patient was followed closely by pediatric endocrinology and pediatric gastroenterology, and once her glucose was optimized, her transaminitis and lactic acidosis resolved. Although an uncommon condition, it is important to consider glycogenic hepatopathy in diabetic patients with poor glucose control, abdominal pain, transaminitis and lactic acidosis.

**Topics:**

Diabetic ketoacidosis, hepatopathy, lactic acidosis, transaminitis, glycogen, diabetes mellitus. Brown D, et al. A Case Report of Glycogenic Hepatopathy. JETem 2021. 6(3):V1–3

**Figure f1-jetem-6-3-v1:**
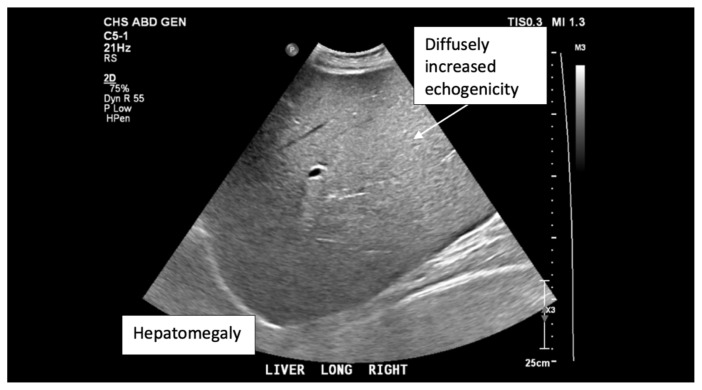


**Figure f2-jetem-6-3-v1:**
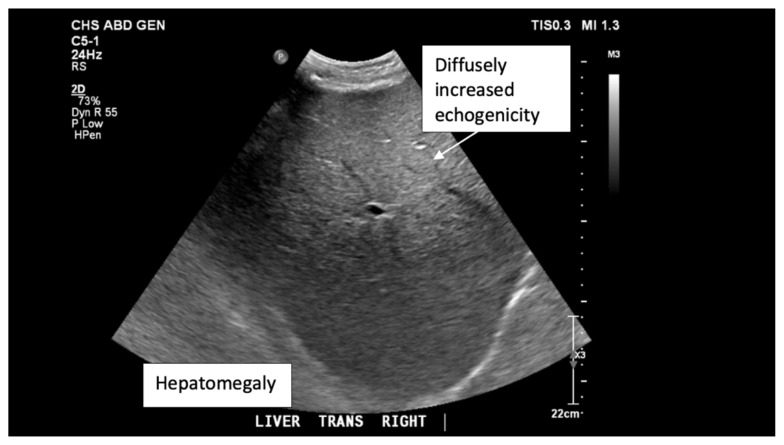


## Brief introduction

[Fig f1-jetem-6-3-v1][Fig f2-jetem-6-3-v1]Glycogenic hepatopathy is a rare condition in which poorly controlled diabetes can lead to excess glycogen accumulation in the hepatocytes.^1^,^2^,^3^ A diagnosis of glycogenic hepatopathy is made in the setting of hepatomegaly, abdominal pain and elevated liver enzymes. The gold standard of testing is a liver biopsy.^1^,^2^,^3^,^4^,^5^,^6^ Untreated, this condition may lead to liver fibrosis.^3^,^6^

## Presenting concerns and clinical findings

A 15-year-old female presented as a transfer from an outside facility for management of pediatric diabetic ketoacidosis (DKA). The patient had a history of poorly controlled type 1 diabetes mellitus and had been non-adherent to her insulin regimen. She had experienced abdominal pain for one to two months. She had been nauseated and vomiting for 2–3 days before presenting in diabetic ketoacidosis. On the morning of presentation, she was found to have altered mental status and was evaluated at a local emergency department.

On examination, she was drowsy but able to wake and answer questions. She was tachycardic, tachypneic, had dry mucous membranes, and diffuse abdominal tenderness that was worse in the right upper quadrant. Physical examination also revealed hepatomegaly. Laboratory studies revealed persistent diabetic ketoacidosis, lactic acidosis and elevated liver transaminases. She had a pH of 7.06 (reference range 7.32–7.43), an AST of 2,128 (14–36 U/L), an ALT of 841(<35 U/L) and a lactic acid level of 12.5 (0.7–2.0 MMOL/L).

## Significant findings

The ultrasound images reveal hepatomegaly and an increased echogenicity of the liver parenchyma that is diffuse. The increased echogenicity can be best appreciated by a comparison to surrounding structures. It is important to note that the increased echogenicity is non-focal and consistent throughout the entire liver in multiple views. These findings can be consistent with nonalcoholic steatohepatitis as well as glycogenic hepatopathy.

## Patient course

Upon arrival to our facility, laboratory studies were reevaluated, and the patient was noted to be in persistent diabetic ketoacidosis. A hepatitis panel and acetaminophen level were ordered, and both negative. A right upper quadrant ultrasound was performed and revealed increased parenchymal echogenicity that was coarse in appearance. This coarse, diffuse, increased echogenicity was concerning for liver parenchymal disease with the differential diagnosis including fatty infiltration.

The patient was closely monitored in the pediatric intensive care unit. She was continued on insulin infusion and electrolytes were replaced. Chart review revealed previous liver enzymes within normal limits. During the hospitalization, her anion gap closed and her beta-hydroxybutyrate returned to normal and the patient was transitioned to subcutaneous insulin.

Her nausea and abdominal pain improved with her clinical course. Although her lactic acid improved from 12.5 to 4.9MMOL/L (0.7–2MMOL/L), it remained elevated despite intravenous fluid resuscitation and maintenance therapy. Her AST trended from 2128 to 1674 U/L (reference range 14–36 U/L) and her ALT trended from 841 to 728 U/L (<35U/L) upon discharge. Pediatric endocrinology was consulted and assisted with the diagnosis of glycogenic hepatopathy. The patient was stabilized, medically transferred out of the ICU and ultimately discharged home from the hospital to follow with pediatric endocrinology and pediatric gastroenterology.

Chart review for the follow up appointments reveals that the patient worked closely with pediatric endocrinology as well as her primary care provider to adhere to a glucose regimen that allowed her hemoglobin A1C to improve to 8.7% (reference range <6.0%), AST and ALT to normalize and lactic acid return to normal.

## Discussion

The true prevalence of glycogenic hepatopathy is unknown.^1^,^2^,^3^,^6^ The available medical literature primarily consists of multiple case reports. This condition is believed to develop when marked hyperglycemia is combined with supraphysiological amounts of insulin. 1,3 It occurs primarily in patients with type 1 diabetes mellitus. Glycogenic hepatopathy is more common in children, as opposed to adults with metabolic syndrome, who typically develop nonalcoholic fatty liver disease. 2,3 Hepatomegaly can have a rapid onset and rapid resolution with adherence to tightly controlled glucose management.^1^,^2^,^3^ Glycogenic hepatopathy, although uncommon, can lead to liver fibrosis and ultimately to cirrhosis.^1^,^3^ This serious complication illuminates why physicians need to be aware of this uncommon condition in pediatrics.

The diagnosis is made by a liver biopsy as the gold standard.^1^,^2^,^3^,^5^,^6^ A clinical diagnosis can be difficult to make. However, if the patient presents with elevated liver enzymes, hepatomegaly, abdominal pain (secondary to hepatic capsule stretch) and a history consistent with poorly controlled diabetes mellitus or in diabetic ketoacidosis, it points to glycogenic hepatopathy as long as other causes of hepatitis are ruled out.^1^,^2^

Ultrasound findings are not specific to glycogenic hepatopathy and can also occur in nonalcoholic fatty liver disease.^1^ Ultrasound findings include hepatomegaly and a diffusely increased coarse echogenicity.^1^ CT and dual echo MRI can help differentiate between nonalcoholic fatty liver and glycogenic hepatopathy. 1,2,6

Elevated aspartate transaminase and alanine transaminase levels are expected. Lactic acid elevation is common in diabetic ketoacidosis; however, the lactic acidosis is rarely the major contributor to the overall acidotic state.^7^ Therefore, outside of glycogenic hepatopathy, significant lactic acid elevation would be concerning for an underlying hypoperfusion, secondary to hypovolemia, hypotension and hyperventilation.^7^ Hyperglycemia and high levels of insulin can decrease gluconeogenesis and decrease the rate of conversion of pyruvate to glucose. This excess pyruvate lends to production of lactic acid.^2^,^3^ With adherence to an insulin regimen, the patient’s hepatomegaly and transaminitis can resolve within two weeks.^1^,^5^

The primary learning point for this case is to consider glycogenic hepatopathy in the setting of persistent lactic acidosis, especially in a pediatric patient with uncontrolled diabetes mellitus or DKA. A timely diagnosis of glycogenic hepatopathy may decrease unnecessary testing, lead to decreased length of ICU stay and shorter hospitalizations with improved outcomes for the patient.

## Supplementary Information








