# Supplementary figures and images for: A Case Report of Glycogenic Hepatopathy
Source: J Educ Teach Emerg Med. 2021 Jul 15;6(3):V1–3. doi: 10.21980/J8SQ0Z (PMC10332688; doi:10.21980/J8SQ0Z)

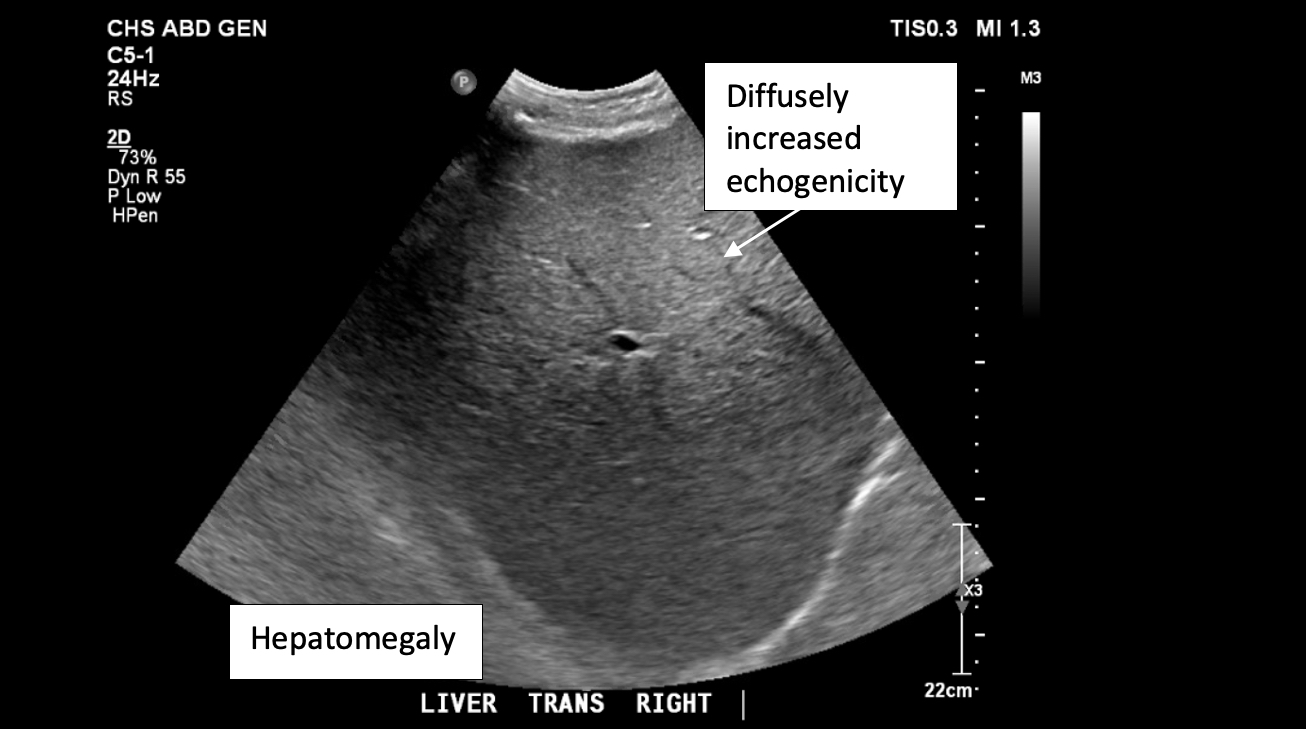

Supplement: Supplementary file 1 [file jetem-6-3-v1-supp1.jpeg]

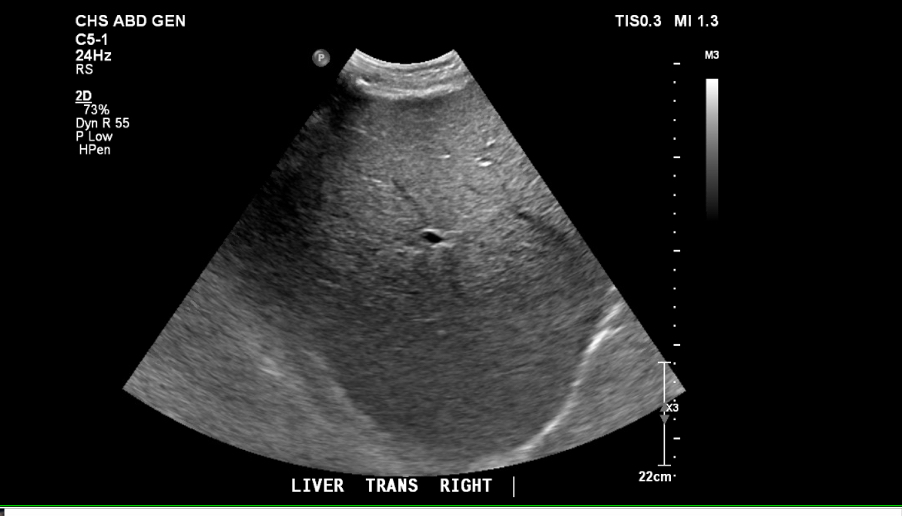

Supplement: Supplementary file 2 [file jetem-6-3-v1-supp2.jpeg]

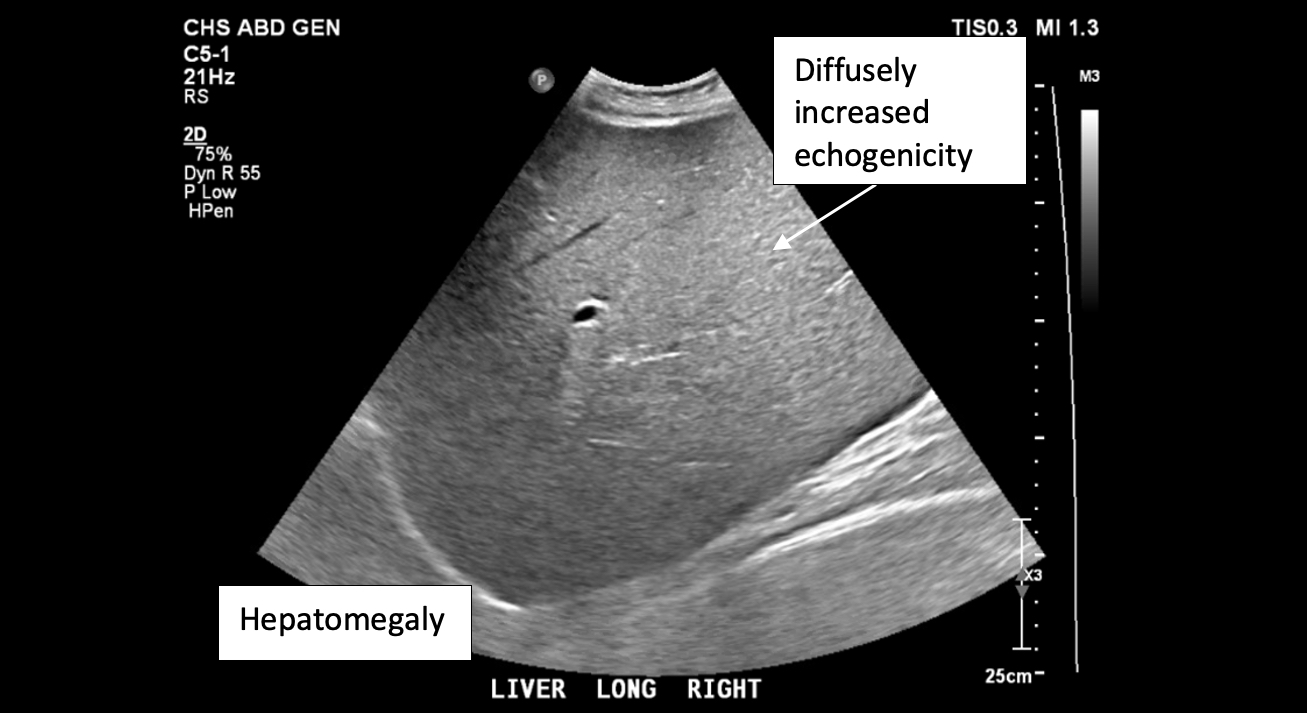

Supplement: Supplementary file 3 [file jetem-6-3-v1-supp3.jpeg]

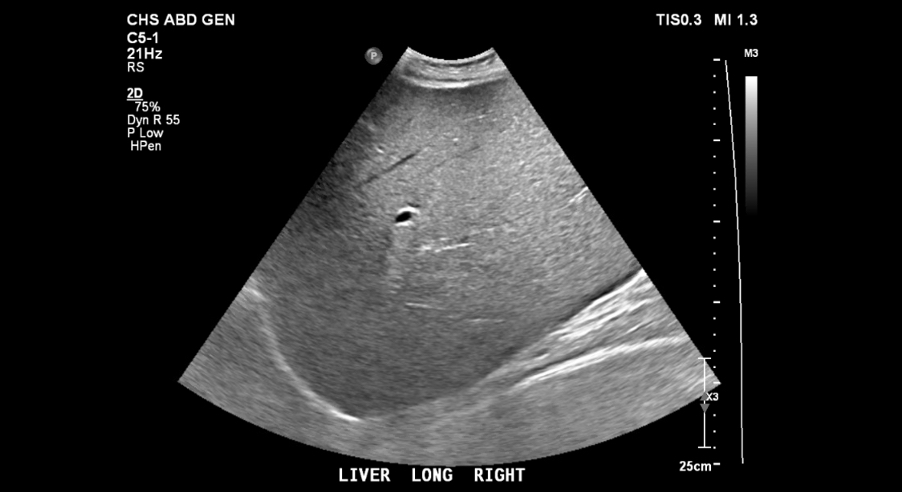

Supplement: Supplementary file 4 [file jetem-6-3-v1-supp4.jpeg]
